# Molecular evolution of the capsid (*VP1*) region in human norovirus genogroup II genotype 3

**DOI:** 10.1016/j.heliyon.2020.e03835

**Published:** 2020-05-03

**Authors:** Mariko Saito, Hiroyuki Tsukagoshi, Hirotaka Ishigaki, Jumpei Aso, Haruyuki Ishii, Kaori Okayama, Akihide Ryo, Taisei Ishioka, Makoto Kuroda, Nobuhiro Saruki, Kazuhiko Katayama, Hirokazu Kimura

**Affiliations:** a Gunma Prefectural Institute of Public Health and Environmental Sciences, 378 Kamioki-machi, Maebashi-shi, Gunma 371-0052, Japan; b Department of Health Science, Gunma Paz University Graduate School of Health Sciences, 1-7-1 Tonyamachi, Takasaki-shi, Gunma 370-0006, Japan; c Kyorin University Hospital, 6-20-2 Shinkawa, Mitaka-shi, Tokyo 181-8611, Japan; d Department of Microbiology, Yokohama City University School of Medicine, 3-9 Fukuura, Yokohama-shi, Kanagawa 236-0004, Japan; e Pathogen Genomics Center, National Institute of Infectious Diseases, 1-23-1 Toyama, Shinjuku-ku, Tokyo 162-8640, Japan; f Laboratory of Viral Infection I, Kitasato Institute for Life Sciences Graduate School of Infection Control Sciences, Kitasato University, 5-9-1 Shirokane, Minato-ku, Tokyo 108-8641, Japan

**Keywords:** Bioinformatics, Microbiology, Virology, Viral disease, Viral genetics, Norovirus, GII.3, Molecular evolution, VP1

## Abstract

Norovirus GII.3 has been suggested to be a prevalent genotype in patients with acute gastroenteritis. However, the genetic properties of the *VP1* region encoding the major GII.3 antigen remain unclear. Here, we performed molecular evolutionary analyses of the GII.3 *VP1* region detected in various countries. We performed time-scaled phylogenetic analyses, selective pressure analyses, phylogenetic distance analyses, and conformational epitope analyses. The time-scaled phylogenetic tree showed that an ancestor of the GII.3 *VP1* region diverged from the common ancestors of the GII.6, GII.11, GII.18, and GII.19 approximately 70 years ago with relatively low divergence. The evolutionary rate of the GII.3 *VP1* region was rapid (4.82 × 10^−3^ substitutions/site/year). Furthermore, one positive site and many negative selection sites were observed in the capsid protein. These results suggest that the GII.3 *VP1* region rapidly evolved with antigenic variations.

## 1 Introduction

Noroviruses (NoVs) are nonenveloped RNA viruses belonging to the family *Caliciviridae* and genus *Norovirus*. Numerous previous reports have indicated that NoV is a major causative agent of acute gastroenteritis in humans of all ages (Patel et al., 2008; Robilotti et al., 2015; Mans, 2019). Indeed, NoV was responsible for >90% of patients with viral gastroenteritis, rendering this infection a major disease burden worldwide (Patel et al., 2008).

The NoV genome length is approximately 7.6 kb, comprising a single plus-strand RNA; the genome has three open reading frames (ORFs) and encodes eight proteins (Robilotti et al., 2015). ORF1 encodes six nonstructural proteins, including a protease and RNA-dependent RNA polymerase, whereas ORF2 encodes a capsid protein, VP1, as the major antigen (Robilotti et al., 2015). Moreover, ORF3 encodes another capsid protein, VP2, although its function remains unclear (Green, 2013). A previous report showed that NoV is classified into two genogroups [genogroup I (GI) and GII], which are detailed assigned to over 30 genotypes (GI.1–GI.9 and GII.1–GII.22) (Kroneman et al., 2013; Vinjé, 2015). Among them, compared with NoV GI viruses, NoV GII viruses are more dominantly detected in patients with acute gastroenteritis (Patel et al., 2008; Green, 2013). Moreover, some genotypes of GII viruses, such as GII.3, GII.4, GII.6, and GII.7, are frequently detected in these patients (Green, 2013). In particular, the genotype GII.4 suddenly emerged during the 2006/07 season and caused a pandemic of gastroenteritis worldwide (Tu et al., 2007; Motomura et al., 2008; Siebenga et al., 2009; Lam et al., 2012). Some previous reports have suggested that GII.3 is the predominant genotype in infants and children (Boon et al., 2011; Wangchuk et al., 2017; Boonchan et al., 2018; Liu et al., 2018). Indeed, GII.3 was the most frequently detected virus in children with gastroenteritis in Japan during 2003–2004 (Phan et al., 2006). To date, several molecular epidemiology and molecular evolutionary studies regarding certain prevalent genotypes, such as GII.2, GII.4, and GII.17, have been reported (Motoya et al., 2017; Parra et al., 2017; Siqueira et al., 2017; Mizukoshi et al., 2017; Nagasawa et al., 2018); however, studies investigating GII.3 evolution in detail are scarce. Here, to understand the characteristics of GII.3 evolution, we performed a molecular evolutionary study of the *VP1* region of GII.3 detected in various countries.

## 2 Materials and methods

### 2.1 Strains

The full-length NoV *VP1* nucleotide coding region were collected from GenBank in November 11, 2019. The collected sequences were assigned using a NoV genotyping tool (Kroneman et al., 2011), and the GII.3 genotype strains were selectively collected. Among them, those with ambiguous sequences and an unknown year of collection were omitted from the data. Moreover, the Recombination Detection Program v. 4.95 as previously described (7 methods, BOOTSCAN/RECSCAN, CHIMAERA, GENECONV, MAXCHI, RDP, SISCAN, and 3SEQ) was used for recombination analysis (Martin et al., 2015). Significance of the *p*-value was less than 0.001. Recombinant was determined when they were confirmed by > four of these methods. However, no recombinant regions were found. In this study, we used 239 full-length of the GII.3 *VP1* sequences in the dataset (Table S1).

### 2.2 Time-scaled phylogeny and estimation of the rate of evolution using the Bayesian Markov chain Monte Carlo (MCMC) method

To perform the molecular evolutionary analysis of the present strains, we constructed phylogenetic trees of the NoV *VP1* region using the MCMC method. Evolutionary dynamics of the molecular clock was examined by the MCMC method in the BEAST package v2.4.8 (Suchard et al., 2001; Drummond and Rambaut, 2007; Bouckaert et al., 2014). To make the accurate phylogenetic tree, we further used the all GII genotypes sequences, including porcine NoV GII (GII.11, GII.18) and other human NoV genogroup II genotypes (19 strains), and an outgroup strain human NoV genogroup I genotype (GI.1) in the present dataset (total 261 strains in Table S1). First, we used the suitable substitution model using the jModelTest 2.1.10 program (Guindon and Gascuel, 2003; Darriba et al., 2012). Thereafter, the best of 4 clock models (strict clock, relaxed clock exponential, relaxed clock log normal, and random local clock) and 2 tree prior models (coalescent constant population and coalescent exponential population) were estimated using the path-sampling/stepping stone-sampling marginal likelihood estimation method. Finally, the optimal dataset was estimated as the relaxed clock exponential and exponential tree prior models. The MCMC chains were 250,000,000 steps with sampling every 5,000 steps. The convergence of all parameters (Effective Sample Size>200), through inspection with Tracer v1.6. After discarding the 10% burn-in, phylogenetic trees were generated with TreeAnnotator v2.4.8 and visualized with FigTree v1.4.0. The credibility of each branch was supported by the 95% highest posterior density (HPD). In addition, the evolution rates of GII.3 were assumed by suitable models selected for each dataset as described above.

### 2.3 Calculation of the phylogenetic distance

To calculate the phylogenetic distance, we constructed phylogenetic tree of all GII.3 strains based on the maximum likelihood method using MEGA 7.0 software (Kumar et al., 2016). jModelTest 2.1.10 was used to select the most suitable evolutionary model. We calculated the phylogenetic distances in the present phylogenetic trees using Patristic program (Fourment and Gibbs, 2006).

### 2.4 Creation of the capsid protein structure and estimation of conformational epitopes for the B-cell in the VP1 protein

The NoV GII.3 VP1 dimer structural model of Hu/NoV/GII.3/Toronto 24/1991/CA (GenBank accession no. U02030) was created by MODELLER v9.20 (Webb and Sali, 2014). The templates for homology modeling were made by the crystal structures of 7 strains (PDB ID: 1IHM, 5F4M, 4RPD, 3PUM, 3PA1, 4RPB, and 3SEJ). The capsid structure of GI (PDB ID: 1IHM) was used as a template to construct a shell domain in whole capsid protein (VP1) structures. The sequences of amino acid of each strain were aligned using MAFFTash (Katoh et al., 2002; Standley et al., 2007). The created structures were minimized using the GROMOS96 (van Gunsteren et al., 1996), included by Swiss PDB Viewer v4.1 (Guex and Peitsch, 1997), and predicted by Ramachandran plots via an available server RAMPAGE (Lovell et al., 2003). The final model was made/colored using Chimera v1.13.1 (Pettersen et al., 2004). BEPro (Sweredoski and Baldi, 2008), DiscoTope 2.0 (Kringelum et al., 2012), EPSVR (Liang et al., 2010), and EPCES (Liang et al., 2007) were employed for the prediction of conformational B-cell epitopes for the capsid VP1 protein of the GII.3 strain (Hu/NoV/GII.3/Toronto 24/1991/CA) with cut-off values of the epitopes were set at 1.3 (BEPro), −3.7 (DiscoTope 2.0), and 70 (EPSVR, EPCES). Consensus sites in the 4 tools and regions with close residues more than 2 of the sites on the dimer structures of capsid protein were estimated as conformational epitopes.

### 2.5 Positive and negative selection analyses

The rates of synonymous (*dS*) and non-synonymous (*dN*) substitution at each codon were estimated by the Datamonkey server to assume the selective sites in the capsid protein coding (*VP1*) region of GII.3 (Pond and Frost, 2005; Delport et al., 2010). We used FEL, IFEL, and SLAC methods and estimated negative (*dN* < *dS*) and positive (*dN* > *dS*) selection sites. A significance level was *p* < 0.05. The two-tail extended binomial distribution (SLAC method) was applied to estimate the *p*-value. The single-degree-of-freedom likelihood ratio test (FEL and IFEL methods; the chi-squared asymptotic distribution was used) to estimate a site as positive or negative selection.

### 2.6 Estimation of the similarities

Similarities between the nucleotide sequences and Hu/NoV/GII.3/Toronto 24/1991/CA were estimated by an available software (SimPlot program 3.5.1) as previously descrived (Lole et al., 1999). A reference strain Hu/NoV/GII.3/Toronto 24/1991/CA was used as the query sequence. The similarity was examined using the Kimura 2-parameter method with window size of 200 nucleotides in length and a step size of 20 nucleotides in the full-length *VP1* coding region.

### 2.7 Phylodynamics based on the Bayesian skyline plots (BSP)

To examine the phylodynamics of the GII.3 strains based on BSP analyses were made by BEAST v2.4.8. as previously described (Suchard et al., 2001; Drummond and Rambaut, 2007; Bouckaert et al., 2014). The best clock and substitution models for these analyses were estimated as described above. Visualization of the analyzed plots were made by the 95% HPD with Tracer.

### 2.8 Statistical analysis

The Kruskal-Wallis test was applied to determine significant differences between the clusters for the evolutionary rates and phylogenetic distance.

## 3 Results

### 3.1 Phylogeny and evolution rates of the capsid protein (VP1) coding region in NoV GII.3

**Figure 1:**
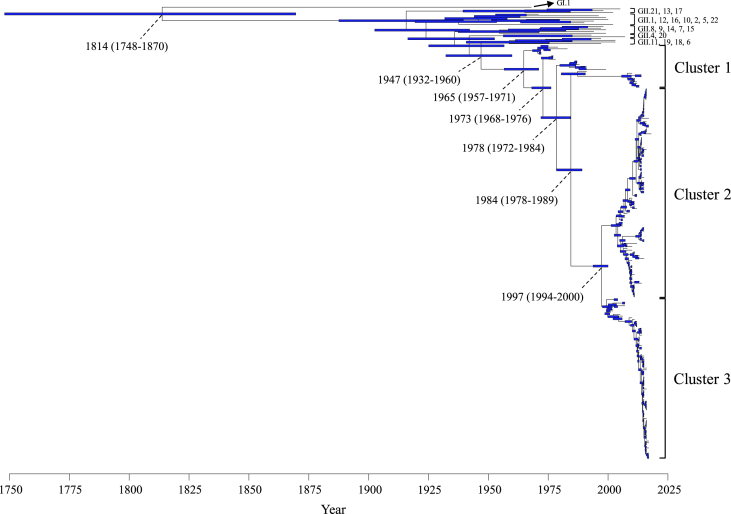
Time-scaled phylogenetic tree for the GII.3 *VP1* region constructed using the Bayesian Markov chain Monte Carlo method. The scale bar represents time (years). Blue bars indicate the 95% highest posterior density for the branched year.

To make time-scale evolutionary analysis, we created a time-scaled phylogenetic tree based on the MCMC method. The tree showed that the *VP1* region is divided into three clusters (Figure 1). The most recent common ancestor of the GII.3 strains was assumed at 1947 (95% HPD, 1932–1960) and formed three clusters after 1965 (95% HPD, 1957–1971). Cluster 1 emerged in 1965 (95% HPDs, 1957–1971). Subsequently, clusters 2 and 3 emerged in 1997 (95% HPD, 1994–2000). The evolutionary rate of the *VP1* region in Gll.3 strains was estimated as 4.82 × 10^−3^ substitutions/site/year (95% HPD, 4.14–5.48 × 10^−3^ substitutions/site/year). The nucleotide substitution rates showed a significant difference among each cluster in GII.3 (Kruskal–Wallis test; *p* < 0.001). Comparing the evolutionary rates among the clusters, that of cluster 2 was the most rapid estimated at 5.29 × 10^−3^ substitutions/site/year (95% HPD, 4.29–6.34 × 10^−3^ substitutions/site/year) (Table S2).

### 3.2 Phylogenetic distances (p-distance) of the VP1 region in the GII.3 strains

**Figure 2:**
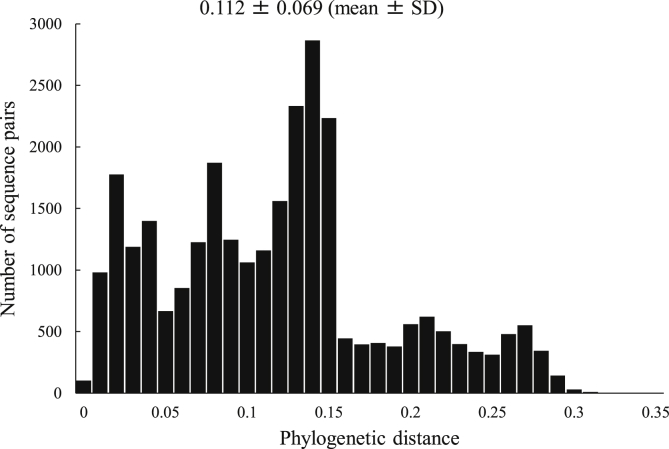
Phylogenetic distance of intra-genotypes in GII.3 strains. The y-axis represents the number of sequence pairs corresponding to each distance. The x-axis shows phylogenetic distances.

We estimated the phylogenetic distance (p-distance) of the *VP1* region in the GII.3 strains. The p-distance value of all GII.3 strains was 0.112 ± 0.069 (mean ± standard deviation, SD) (Figure 2). The mean values of each cluster were within the range 0.029–0.116, and the p-distances were significantly different among GII.3 clusters. Cluster 1 had the largest phylogenetic distance [0.116 ± 0.061 (mean ± SD)].

### 3.3 Mapping of conformational epitopes and positive selection sites on the capsid (VP1) protein structures of GII.3

**Figure 3:**
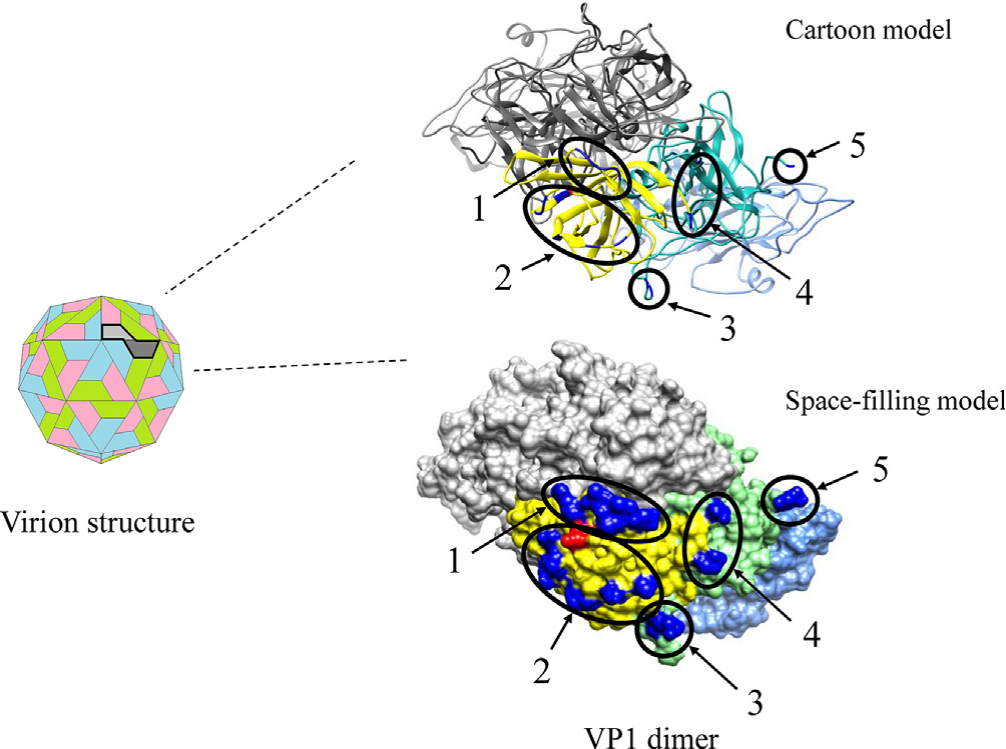
Structural models for the capsid VP1 protein of GII.3. The virion structure and three-dimensional VP1 dimer structures (cartoon and space-filling models) for the Hu/NoV/GII.3/Toronto 24/1991/CA strain are shown. Chains comprising the dimer structures are colored as follows: yellow: P2 domain (chain A); green: P1 domain (chain A); light blue: shell domain (chain A); gray: chain B; blue: predicted epitopes (chain A); red: positive selection site (aa385).

To comprehensively estimate between the conformational epitopes on the GII.3 VP1 protein and positive/selection sites, we mapped them in the protein using *in silico* methods (Figure 3). Five sites were estimated as conformational epitopes. Of them, 2 epitopes were located in the exterior surface of the P2 domain. Moreover, several amino acid substitutions were estimated on and/or around these epitopes.

One positively selected site (aa385) was estimated as follows: Gly385Asp, Ser, His, Asp385Gly, Ser, and Ser385Gly, Asp. This was located in the P2 domain, while many negatively selected sites were estimated (257 sites).

### 3.4 Similarity plot analysis of the VP1 region in the present GII.3 strains

**Figure 4:**
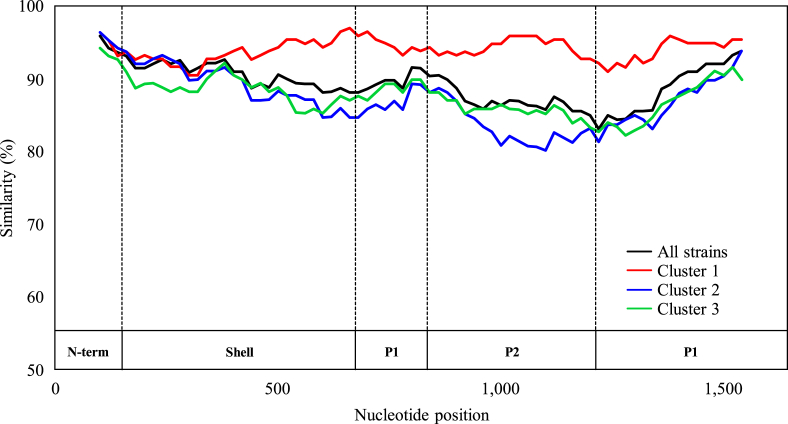
Similarity plot analysis of the *VP1* region across GII.3 strains. Nucleotide similarity to the Hu/NoV/GII.3/Toronto 24/1991/CA strain was calculated using SimPlot analysis. Each cluster similarity to the Hu/NoV/GII.3/Toronto 24/1991/CA is represented. The positions of the shell, P1, and P2 domains in *VP1* region are shown below the graph.

Using SimPlot, we performed similarity plot analysis based on the full-length *VP1* region (Figure 4). The results revealed that the P1 and P2 domains yielded more divergence of the *VP1* region in the present GII.3. In cluster 2 of the GII.3 strains, the similarities between the P1 and P2 domains (80.1%–91.5%) were lower than those that occurred in the shell domain (84.7%–94.2%).

### 3.5 Phylodynamics of the GII.3 VP1 coding regions

**Figure 5:**
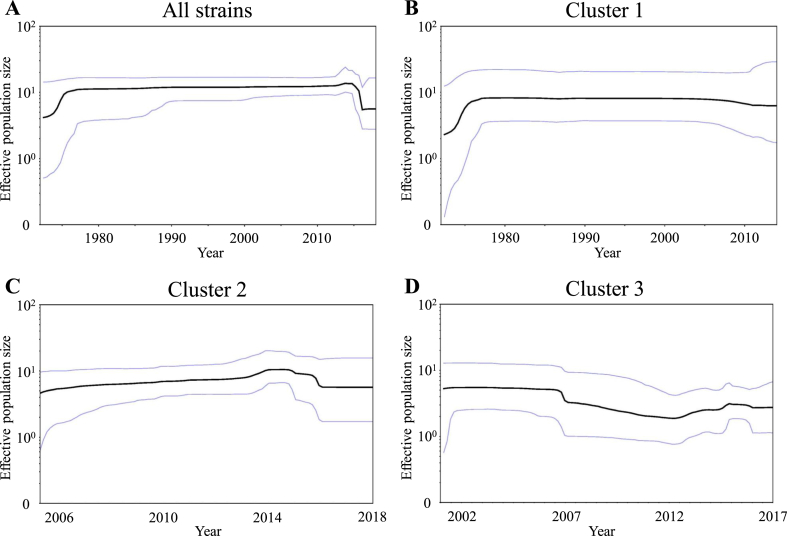
Bayesian skyline plot for the VP1 sequences of GII.3. Plots for all GII.3 strains (A), cluster 1 (B), cluster 2 (C), and cluster 3 (D) are shown. The y-axis represents the effective population size on the logarithmic scale, whereas the x-axis denotes the time in years. The solid black line indicates the median posterior value. The intervals with the highest posterior densities (95%) are indicated by blue lines.

To estimate the changes of the phylodynamics of the GII.3, we calculated the effective population sizes (EPS) using the BSP method. The EPS values of the *VP1* region in the GII.3 strains constant up to 2013. Thereafter, the EPS slightly dropped (Figure 5). In cluster 3 of the GII.3 strains, the mean EPS gradually reduced since 2007, although the EPS slightly increased by 2012. No changes in the mean EPS of the other sample clusters were observed.

## 4 Discussion

In this study, we performed detailed molecular evolutionary analyses of the GII.3 *VP1* region detected in various countries. Thus, an ancestor of the GII.3 *VP1* region diverged from the common ancestors of the GII.6, GII.11, GII.18, and GII.19 strains approximately 70 years ago and formed several clusters with relatively low genetic divergence in the time-scaled phylogenetic tree (Figure 1). The evolution rates of the GII.3 *VP1* coding region was estimated at 4.82 × 10^−3^ substitutions/site/year. Furthermore, one positive site and many negative selection sites were found in the GII.3 capsid protein. These results suggest that the GII.3 *VP1* region rapidly evolved and had antigenic variations.

The present phylogenetic tree showed that a common ancestor of the GII.3 *VP1* region diverged at around 70 years ago and further diverged at 1984 and 1997, resulting in the formation of three clusters (Figure 1). The common ancestor of this genotype was emerged after another major genotype, GII.4, while GII.3 emerged prior to GII.2 (Figure 1) (Motoya et al., 2017; Mizukoshi et al., 2017; Nagasawa et al., 2018). Previous studies have also suggested that the GII.3 *VP1* region is phylogenetically classified into three major clusters (clusters I to III) (Boon et al., 2011; Mahar et al., 2013), a finding that is compatible with the present data. However, the present strains belonging to cluster 1 correspond to clusters I and II, while the present strains assigning to clusters 2 and 3 correspond to cluster III (Boon et al., 2011; Mahar et al., 2013). These results suggest that the GII.3 strain continuously evolved and adapted to humans.

In the present study, the evolutionary rate of the GII.3 *VP1* region collected from various countries was assumed at 4.82 × 10^−3^ substitutions/site/year. Previous studies have reported the evolutionary rates in some genotypes of the NoV *VP1* region (Boon et al., 2011; Mahar et al., 2013; Kobayashi et al., 2016; Motoya et al., 2017; Parra et al., 2017; Mizukoshi et al., 2017; Nagasawa et al., 2018). For example, Boon et al. showed that the evolutionary rate of 7 GII.3 strains detected in the USA and 56 GII.3 strains available in GenBank was 4.16 × 10^−3^ substitutions/site/year (Boon et al., 2011). Moreover, another report estimated that the rate of evolution in 6 GII.3 strains collected from Australian children and 66 GII.3 strains from GenBank was 4.16 × 10^−3^ to 6.97 × 10^−3^ substitutions/site/year (Mahar et al., 2013). Current data was similar to previous reports, while there were some differences in the sequence numbers, methodology, and collection date (Boon et al., 2011; Mahar et al., 2013). These may reflect the differences in evolutionary rates between the previous (3.76 × 10^−3^ substitutions/site/year; Kobayashi et al., 2016) and present data. In addition, the evolutionary rate of the *VP1* region in another major prevalent type GII.4, which was detected in various countries, was estimated at approximately 7.7 × 10^−3^ substitutions/site/year (Motoya et al., 2017), and this value was higher than that in the GII.3 strain. Moreover, genetic divergence (phylogenetic distance) of the GII.3 *VP1* region was shorter than that of GII.4 (0.112 ± 0. 069 vs. 0.210 ± 0.105) (Motoya et al., 2017). These results suggested that the divergence of the GII.3 *VP1* coding region may be relatively low.

Next, we analyzed conformational epitopes and mapped them on the GII.3 capsid protein (Figure 3). Previous reports have suggested that most epitopes were estimated in the P2 domain (Motoya et al., 2017; Nagasawa et al., 2018). Motoya et al. showed that four epitopes were found in the GII.4 P2 domain (Motoya et al., 2017). Moreover, Nagasawa et al. showed that epitopes were mainly found in the GII.2 P2 domain (Nagasawa et al., 2018). These reports also suggested that the differences in the amino acid substitutions may reflect differences in the antigenicity of these genotypes (Motoya et al., 2017; Nagasawa et al., 2018). In this study, we showed that the GII.3 strain includes two epitopes in the P2 domain and three epitopes in the P1 domain (Figure 3). Among them, aa385 in the P2 domain was estimated as a positive selection site (Gly385Asp, Ser, His, Asp385Gly, Ser, and Ser385Gly, Asp). This site may act as a surface-exposed residue, resulting in a pivotal binding site of a neutralizing antibody (Mahar et al., 2013). Thus, it is possible that the positive selection site plays important roles in the changes in the GII.3 capsid protein antigenicity (Boon et al., 2011; Mahar et al., 2013, 2014).

Moreover, we performed phylodynamic analysis of the GII.3 *VP1* region (Figure 5). Overall, phylodynamic fluctuations of the gene was minimal. However, in cluster 3 of the GII.3 strains, the size was slightly increased by 2012. Very recent molecular epidemiological studies have reported outbreaks of gastroenteritis due to the GII.3 strain (Boonchan et al., 2018; Kim et al., 2018; Liu et al., 2018). Thus, the fluctuation of cluster 3 may reflect the transient prevalence of the GII.3 strain (Kim et al., 2018).

Recent studies have suggested that NoV pandemics may be associated with recombination between ORF1 and ORF2, as well as with the properties of the RNA-dependent RNA polymerase (RdRp) (Bull et al., 2010; Eden et al., 2013; Ozaki et al., 2018). In this study, we only performed the molecular evolutionary analysis in the GII.3 *VP1* region. Previous reports showed that the recombination between NoV ORF1 (including the *RdRp* region) and ORF2 (including the *VP1* region) resulted in the emergence of the distinct *RdRp* genotype of NoV (Bidalot et al., 2017; Lu et al., 2017; Nagasawa et al., 2018; Niendorf et al., 2017; Tohma et al., 2017). Furthermore, the *VP1* region co-evolved with the *RdRp* region (Mizukoshi et al., 2017; Ozaki et al., 2018). Therefore, the RdRp protein may affect the evolutionary process of the capsid proteins (VP1 and VP2 proteins) (Mizukoshi et al., 2017). To deeply study the molecular evolutionary analyses of the NoV, the analyses of both the *RdRp* and *VP1* regions may be considered (Mizukoshi et al., 2017).

In conclusion, NoV GII.3 strains are frequently detected following other genotypes GII.4 and GII.2. Although there were some sporadic cases where GII.3 did not cause pandemics of acute gastroenteritis like GII.4 (Bull et al., 2010; White, 2014). However, whether the prevalence of the GII.3 is in the near future may not be known yet. Therefore, continuous molecular epidemiological and molecular evolutionary analyses of GII.3 may be required.

## Declarations

### Author contribution statement

Mariko Saito, Hirokazu Kimura: Conceived and designed the experiments; Performed the experiments; Analyzed and interpreted the data; Contributed reagents, materials, analysis tools or data; Wrote the paper.

Hiroyuki Tsukagoshi: Performed the experiments; Analyzed and interpreted the data; Wrote the paper.

Hirotaka Ishigaki, Jumpei Aso, Kaori Okayama, Nobuhiro Saruki: Performed the experiments.

Haruyuki Ishii, Kazuhiko Katayama: Conceived and designed the experiments.

Akihide Ryo, Taisei Ishioka: Analyzed and interpreted the data.

Makoto Kuroda: Conceived and designed the experiments; Contributed reagents, materials, analysis tools or data.

### Funding statement

This work was supported by a commissioned project for Research on Emerging and Re emerging Infectious Diseases from the 10.13039/100009619Japan Agency for Medical Research and Development, AMED (grant number JP20fk0108120h0401).

### Competing interest statement

The authors declare no conflict of interest.

### Additional information

No additional information is available for this paper.

## Appendix A Supplementary data

Supplementary Tables_20200123.docx Supplementary Tables_20200123.docx
