# Supplementary material for: Molecular evolution of the capsid (VP1) region in human norovirus genogroup II genotype 3
Source: Heliyon. 2020 May 3;6(5):e03835. doi: 10.1016/j.heliyon.2020.e03835 (PMC7205756; doi:10.1016/j.heliyon.2020.e03835)
Supplement: Supplementary Tables_20200123.docx [file mmc1.docx]

Supplementary Data

**Supplementary Tables**

**Supplementary Table S1.** Strains used in this study (Genotypes other than GII.3).

| GenBank accession No. | Strain | Collected year | Country | Genotypes | References or authorship |
| --- | --- | --- | --- | --- | --- |
| M87661 | Hu/GI.1/Norwalk/1968/US | 1968 | USA | GI.1 | 1 |
| U07611 | Hu/GII.1/Hawaii/1971/US | 1971 | USA | GII.1 | 2 |
| X81879 | Hu/GII.2/Melksham/1994/UK | 1994 | UK | GII.2 | 3 |
| X76716 | Hu/GII.4/Bristol/1993/UK | 1993 | UK | GII.4 | 4 |
| AJ277607 | Hu/GII.5/Hillingdon/1990/UK | 1990 | UK | GII.5 | 5 |
| AB039778 | Hu/GII.6/Saitama/U16/1997/JP | 1997 | Japan | GII.6 | 6 |
| AF414409 | Hu/GII.7/Gwynedd/273/1994/US | 1994 | USA | GII.7 | 7 |
| AB039780 | Hu/GII.8/Saitama/U25/1998/JP | 1998 | Japan | GII.8 | 6 |
| AY038599 | Hu/GII.9/VA97207/1997/USA | 1997 | USA | GII.9 | 8 |
| AF427118 | Hu/GII.10/Erfurt/546/2000/DE | 2000 | Germany | GII.10 | Kuenkel, U., et al |
| AB074893 | Sw/GII.11/Sw918/1997/JP | 1997 | Japan | GII.11 | 9 |
| AB032758 | Hu/GII.12/Aichi/1996/JP | 1996 | Japan | GII.12 | 10 |
| AY113106 | Hu/GII.13/Fayetteville/1998/US | 1998 | USA | GII.13 | Vinje, J., et al |
| AY130761 | Hu/GII.14/M7/1999/US | 1999 | USA | GII.14 | Vinje, J. |
| AY130762 | Hu/GII.15/J23/1999/US | 1999 | USA | GII.15 | Vinje, J. |
| AY502010 | Hu/GII.16/Tiffin/1999/USA | 1999 | USA | GII.16 | 11 |
| AY502009 | Hu/GII.17/CS-E1/2002/USA | 2002 | USA | GII.17 | 11 |
| AY823304 | Sw/GII.18/OH-QW101/2003/US | 2003 | USA | GII.18 | 12 |
| AY823306 | Sw/GII.19/OH-QW170/2003/US | 2003 | USA | GII.19 | 12 |
| AB542917 | Hu/GII.20/OC07118/2007/JP | 2007 | Japan | GII.20 | 13 |
| AB542915 | Hu/GII.21/OC05024/2005/JP | 2005 | Japan | GII.21 | 13 |
| AB083780 | Hu/GII.21/OC05024/2005/JP | 2005 | Japan | GII.22 | Kuzuguchi, T., et al |

**Supplementary Table S1** (continued). Strains used in this study (GII.3 genotype).

| GenBank accession No. | Strain | Collected year | Country | Cluster | References or authorship |
| --- | --- | --- | --- | --- | --- |
| AY030312 | Hu/NLV/GII/MD101-2/1987/US | 1987 | USA | 1 | 14 |
| AY030313 | Hu/NLV/GII/MD134-10/1987/US | 1987 | USA | 1 | 14 |
| DQ379713 | Hu/GII/Goulburn Valley G5175 A/1983/AUS | 1983 | Australia | 1 | 15 |
| HM072040 | Hu/GII.3/CHDC5365/1991/US | 1991 | USA | 1 | 16 |
| HM072041 | Hu/GII.3/CHDC5261/1991/US | 1991 | USA | 1 | 16 |
| HM072042 | Hu/GII.3/CHDC4671/1979/US | 1979 | USA | 1 | 16 |
| HM072044 | Hu/GII.3/CHDC4031/1988/US | 1988 | USA | 1 | 16 |
| HM072045 | Hu/GII.3/CHDC2005/1975/US | 1975 | USA | 1 | 16 |
| HM072046 | Hu/GII.3/CHDC32/1976/US | 1976 | USA | 1 | 16 |
| JN565063 | Hu/GII.3/Milwaukee009/2010/USA | 2010 | USA | 1 | Vega, E., et al |
| JN699040 | Hu/GII.3/HK54/CN/1977 | 1977 | China | 1 | 17 |
| JQ743333 | Hu/GII.3/1999 | 1999 | USA | 1 | 18 |
| JX846924 | Hu/GII.3/HK71/1978/CHN | 1978 | China | 1 | Madupu, R., et al |
| KC597144 | Hu/GII.3/HK46/1977/CHN | 1977 | China | 1 | Madupu, R., et al |
| KF895841 | Hu/GII.P16-GII.3/Smolensk/S12-31/2012/RUS | 2012 | Russia | 1 | 19 |
| KF944110 | Hu/GII.P16-GII.3/Novosibirsk/Nsk-N1659/2011/RUS | 2011 | Russia | 1 | 19 |
| KF944111 | Hu/GII.P16-GII.3/Novosibirsk/Nsk-N1648/2011/RUS | 2011 | Russia | 1 | 19 |
| KT779557 | Hu/GII.P16-GII.3/Omsk/O1370/2012/RUS | 2012 | Russia | 1 | 19 |
| KY442319 | Hu/US/1972/GII.Pg_GII.3/ShippensburgB24 | 1972 | USA | 1 | 20 |
| KY442320 | Hu/US/1972/GII.Pg_GII.3/ShippensburgC2 | 1972 | USA | 1 | 20 |
| MH702287 | Hu/BT/2013/GII.P16-GII.3/ETR-NV-388 | 2013 | Bhutan | 1 | Pham, A.H., et al |
| MK396772 | Hu/NP/2013/GII.P16-GII.3/ETR-NV-097 | 2013 | Nepal | 1 | Pham, A.H., et al |
| MK762640 | Hu/US/2014/GII.P16-GII.3/Sumner0467 | 2014 | USA | 1 | Barclay, L., et al |
| MK764020 | Hu/US/2014/GII.P16-GII.3/Pittsylvania0388 | 2014 | USA | 1 | Barclay, L., et al |
| U02030 | TV24 (Hu/NoV/GII.3/Toronto 24/1991/CA) | 1991 | Canada | 1 | 21 |
| AB385626 | Hu/GII.3/RotterdamP1D0/2006/NL | 2006 | Netherlands | 2 | 22 |
| AB385627 | Hu/GII.3/RotterdamP1D88/2006/NL | 2006 | Netherlands | 2 | 22 |
| AB385641 | Hu/GII.3/RotterdamP8D0/2006/NL | 2006 | Netherlands | 2 | 22 |
| AB385642 | Hu/GII.3/RotterdamP8D31/2006/NL | 2006 | Netherlands | 2 | 22 |
| GQ849127 | Hu/GII.b-GII.3/Sydney740C/2007/AUS | 2007 | Australia | 2 | 23 |
| GU138208 | Hu/GII.P21-GII.3/RUS/Novosibirsk/Nsk-B93/2009 | 2009 | Russia | 2 | 24 |
| JX984948 | Hu/GII.3/GZ2010-L63/Guangzhou/CHN/2010 | 2010 | China | 2 | 25 |
| KC464326 | Hu/GII.3/41/89/2006/AU | 2006 | Australia | 2 | 26 |
| KC464327 | Hu/GII.3/84/46/2007/AU | 2007 | Australia | 2 | 26 |
| KC464328 | Hu/GII.3/693/425/2008/AU | 2008 | Australia | 2 | 26 |
| KC464329 | Hu/GII.3/537/547/2010/AU | 2010 | Australia | 2 | 26 |
| KF306213 | Hu/GII.3/Jingzhou/2013402/CHN | 2013 | China | 2 | 27 |
| KF931324 | Hu/GII.P21-GII.3/RUS/Novosibirsk/Nsk-D59/2009 | 2009 | Russia | 2 | 28 |
| KF944065 | Hu/GII.P21-GII.3/RUS/Novosibirsk/Nsk-N1175/2010 | 2010 | Russia | 2 | 19 |
| KF944119 | Hu/GII.P21-GII.3/RUS/Novosibirsk/Nsk-N1811/2011 | 2011 | Russia | 2 | 19 |
| KF944147 | Hu/GII.P21-GII.3/RUS/Novosibirsk/Nsk-N2300/2011 | 2011 | Russia | 2 | 19 |
| KF944165 | Hu/GII.P12-GII.3/RUS/Novosibirsk/Nsk-N2664/2011 | 2011 | Russia | 2 | 19 |
| KF944166 | Hu/GII.P12-GII.3/RUS/Novosibirsk/Nsk11-N2675/2011 | 2011 | Russia | 2 | 19 |
| KF944179 | Hu/GII.P21-GII.3/RUS/Novosibirsk/Nsk-N2911/2011 | 2011 | Russia | 2 | 19 |
| KF944266 | Hu/GII.P21-GII.3/RUS/Novosibirsk/Nsk-N5071/2012 | 2012 | Russia | 2 | 19 |
| KJ145323 | 13-BG-1/2013/GII.P21/GII.3 | 2013 | Taiwan | 2 | Wu, F.-T., et al |
| KJ184223 | Hu/GII.P21-GII.3/RUS/Novosibirsk/Nsk-N48/2010 | 2010 | Russia | 2 | 19 |
| KJ184256 | Hu/GII.P21-GII.3/RUS/Novosibirsk/Nsk-N2660/2011 | 2011 | Russia | 2 | 19 |
| KJ499443 | GII/Hu/HKG/2013/GII.3/CUHK-NS-218 | 2013 | China | 2 | Chan, M.C.W., et al |
| KJ634708 | Hu/GII.P21-GII.3/RUS/Novosibirsk/Nsk-N4724/2012 | 2012 | Russia | 2 | 19 |
| KM056394 | Hu/GII.3/SW4/2012/TN | 2012 | Tunisia | 2 | Ayouni, S., et al |
| KM198484 | Hu/GII/30212/2009/VNM | 2009 | Viet Nam | 2 | 29 |
| KM198493 | Hu/GII/30468/2010/VNM | 2010 | Viet Nam | 2 | 29 |
| KM198496 | Hu/GII/20419/2010/VNM | 2010 | Viet Nam | 2 | 29 |

**Supplementary Table S1** (continued). Strains used in this study (GII.3 genotype).

| GenBank accession No. | Strain | Collected year | Country | Cluster | References or authorship |
| --- | --- | --- | --- | --- | --- |
| KM198500 | Hu/GII/C2H-20/2011/VNM | 2011 | Viet Nam | 2 | 29 |
| KM198505 | Hu/GII/20460/2010/VNM | 2010 | Viet Nam | 2 | 29 |
| KM198509 | Hu/GII/20479/2010/VNM | 2010 | Viet Nam | 2 | 29 |
| KM198511 | Hu/GII/C2H-24/2011/VNM | 2011 | Viet Nam | 2 | 29 |
| KM198528 | Hu/GII/C2H-25/2011/VNM | 2011 | Viet Nam | 2 | 29 |
| KM198529 | Hu/GII/C2H-27/2011/VNM | 2011 | Viet Nam | 2 | 29 |
| KM198547 | Hu/GII/C2H-47/2011/VNM | 2011 | Viet Nam | 2 | 29 |
| KM198553 | Hu/GII/30381/2010/VNM | 2010 | Viet Nam | 2 | 29 |
| KM198554 | Hu/GII/C2H-48/2011/VNM | 2011 | Viet Nam | 2 | 29 |
| KM198561 | Hu/GII/30303/2009/VNM | 2009 | Viet Nam | 2 | 29 |
| KM198563 | Hu/GII/C2365/2010/VNM | 2010 | Viet Nam | 2 | 29 |
| KM198572 | Hu/GII/20370/2010/VNM | 2010 | Viet Nam | 2 | 29 |
| KM198573 | Hu/GII/C2H-45/2011/VNM | 2011 | Viet Nam | 2 | 29 |
| KM198583 | Hu/GII/20493/2010/VNM | 2010 | Viet Nam | 2 | 29 |
| KM198586 | Hu/GII/30400/2010/VNM | 2010 | Viet Nam | 2 | 29 |
| KM198590 | Hu/GII/C2H-39/2011/VNM | 2011 | Viet Nam | 2 | 29 |
| KP064097 | E2419 | 2008 | France | 2 | 30 |
| KX355506 | Hu/GII.3/20110200/Vietnam | 2011 | Viet Nam | 2 | Lei, S., et al |
| KY210918 | Hu/GII.P21-GII.3/RUS/Novosibirsk/NS16-A294/2016 | 2016 | Russia | 2 | Zhirakovskaya, E. and Tikunova, N. |
| KY210919 | Hu/GII.P21-GII.3/RUS/Novosibirsk/NS16-C32/2016 | 2016 | Russia | 2 | Zhirakovskaya, E. and Tikunova, N. |
| KY767665 | Hu/GII.3/3-34/2015/HNZZ/CHN | 2015 | China | 2 | Zheng, L., et al |
| KY887597 | Hu/UK/2016/GII.P16_GII.3/NOR-2604 | 2016 | UK | 2 | 31 |
| KY887598 | Hu/UK/2016/GII.P16_GII.3/NOR-2610 | 2016 | UK | 2 | 31 |
| KY887606 | Hu/UK/2016/GII.P16_GII.3/NOR-2598 | 2016 | UK | 2 | 31 |
| LN854569 | GII/Hu/NL/2014/GII.21/Groningen | 2014 | Netherlands | 2 | Bavelaar, H., et al |
| MG892076 | Hu/GII.P21-GII.3/RUS/Novosibirsk/Nsk-N3700/2012 | 2012 | Russia | 2 | 19 |
| MG892946 | Hu/GII.P16-GII.3/RUS/Novosibirsk/NS17-A843/2017 | 2017 | Russia | 2 | Zhirakovskaya, E. and Tikunova, N. |
| MG892954 | Hu/GII.P21-GII.3/RUS/Novosibirsk/NS17-A1088/2017 | 2017 | Russia | 2 | Zhirakovskaya, E. and Tikunova, N. |
| MG892955 | Hu/GII.P16-GII.3/RUS/Novosibirsk/NS17-A1112/2017 | 2017 | Russia | 2 | Zhirakovskaya, E. and Tikunova, N. |
| MH218570 | NORO_100_30_06_2014 | 2014 | UK | 2 | 32 |
| MH218572 | NORO_102_02_07_2014 | 2014 | UK | 2 | 32 |
| MH218573 | NORO_103-1_07_07_2014 | 2014 | UK | 2 | 32 |
| MH218574 | NORO_104_07_07_2014 | 2014 | UK | 2 | 32 |
| MH218575 | NORO_105_05_07_2014 | 2014 | UK | 2 | 32 |
| MH218576 | NORO_106_10_07_2014 | 2014 | UK | 2 | 32 |
| MH218577 | NORO_107_11_07_2014 | 2014 | UK | 2 | 32 |
| MH218578 | NORO_108_17_07_2014 | 2014 | UK | 2 | 32 |
| MH218579 | NORO_109_18_07_2014 | 2014 | UK | 2 | 32 |
| MH218580 | NORO_110_06_08_2014 | 2014 | UK | 2 | 32 |
| MH218581 | NORO_111_05_08_2014 | 2014 | UK | 2 | 32 |
| MH218582 | NORO_112_08_08_2014 | 2014 | UK | 2 | 32 |
| MH218583 | NORO_113_04_09_2014 | 2014 | UK | 2 | 32 |
| MH218584 | NORO_114_14_09_2014 | 2014 | UK | 2 | 32 |
| MH218585 | NORO_115_17_09_2014 | 2014 | UK | 2 | 32 |
| MH218586 | NORO_116_26_09_2014 | 2014 | UK | 2 | 32 |
| MH218587 | NORO_117_01_11_2014 | 2014 | UK | 2 | 32 |
| MH218588 | NORO_118_17_11_2014 | 2014 | UK | 2 | 32 |
| MH218589 | NORO_119_27_11_2014 | 2014 | UK | 2 | 32 |
| MH218590 | NORO_120_28_11_2014 | 2014 | UK | 2 | 32 |
| MH218592 | NORO_122_01_12_2014 | 2014 | UK | 2 | 32 |
| MH218593 | NORO_123_02_01_2015 | 2015 | UK | 2 | 32 |
| MH218594 | NORO_124_10_01_2015 | 2015 | UK | 2 | 32 |
| MH218595 | NORO_125_19_01_2015 | 2015 | UK | 2 | 32 |
| MH218596 | NORO_126_17_02_2015 | 2015 | UK | 2 | 32 |

**Supplementary Table S1** (continued). Strains used in this study (GII.3 genotype).

| GenBank accession No. | Strain | Collected year | Country | Cluster | References or authorship |
| --- | --- | --- | --- | --- | --- |
| MH218597 | NORO_127_05_03_2015 | 2015 | UK | 2 | 32 |
| MH218598 | NORO_127-3_24_03_2015 | 2015 | UK | 2 | 32 |
| MH218599 | NORO_128_07_03_2015 | 2015 | UK | 2 | 32 |
| MH218600 | NORO_129_16_03_2015 | 2015 | UK | 2 | 32 |
| MH218601 | NORO_130_25_03_2015 | 2015 | UK | 2 | 32 |
| MH218602 | NORO_131_08_04_2015 | 2015 | UK | 2 | 32 |
| MH218603 | NORO_132_01_05_2015 | 2015 | UK | 2 | 32 |
| MH218604 | NORO_133_26_05_2015 | 2015 | UK | 2 | 32 |
| MH218618 | NORO_147-2_10_12_2015 | 2015 | UK | 2 | 32 |
| MH218630 | NORO_160_14_04_2015 | 2015 | UK | 2 | 32 |
| MH218646 | NORO_177-2_26_01_2016 | 2016 | UK | 2 | 32 |
| MH218653 | NORO_185_28_09_2015 | 2015 | UK | 2 | 32 |
| MH218654 | NORO_186_28_09_2015 | 2015 | UK | 2 | 32 |
| MH218660 | NORO_199_18_11_2015 | 2015 | UK | 2 | 32 |
| MH218668 | NORO_210_16_12_2015 | 2015 | UK | 2 | 32 |
| MH218671 | NORO_213_16_12_2015 | 2015 | UK | 2 | 32 |
| MH218672 | NORO_214_19_12_2015 | 2015 | UK | 2 | 32 |
| MH218675 | NORO_217_23_12_2015 | 2015 | UK | 2 | 32 |
| MH218676 | NORO_218_24_12_2015 | 2015 | UK | 2 | 32 |
| MH218677 | NORO_219_25_12_2015 | 2015 | UK | 2 | 32 |
| MH218678 | NORO_220_28_12_2015 | 2015 | UK | 2 | 32 |
| MH218679 | NORO_221_28_12_2015 | 2015 | UK | 2 | 32 |
| MH218680 | NORO_222_31_12_2015 | 2015 | UK | 2 | 32 |
| MH218681 | NORO_223_01_01_2016 | 2016 | UK | 2 | 32 |
| MH218682 | NORO_224_01_01_2016 | 2016 | UK | 2 | 32 |
| MH218683 | NORO_225_04_01_2016 | 2016 | UK | 2 | 32 |
| MH218686 | NORO_228_11_01_2016 | 2016 | UK | 2 | 32 |
| MH218688 | NORO_230_14_01_2016 | 2016 | UK | 2 | 32 |
| MH218690 | NORO_232_20_01_2016 | 2016 | UK | 2 | 32 |
| MH218693 | NORO_238_17_02_2016 | 2016 | UK | 2 | 32 |
| MH218696 | NORO_34-6_05_05_2015 | 2015 | UK | 2 | 32 |
| MH218712 | NORO_55_15_02_2014 | 2014 | UK | 2 | 32 |
| MH218717 | NORO_63-4_07_10_2015 | 2015 | UK | 2 | 32 |
| MH218732 | NORO_93_03_12_2014 | 2014 | UK | 2 | 32 |
| MH218738 | NORO_99_09_06_2014 | 2014 | UK | 2 | 32 |
| MK773588 | Hu/US/2018/GII.P16-GII.3/Hennepin0275 | 2018 | USA | 2 | Barclay, L., et al |
| MK907787 | G19_016 | 2014 | France | 2 | Strubbia, S., et al |
| MK907798 | G19_034 | 2008 | France | 2 | Strubbia, S., et al |
| AB385634 | Hu/GII.3/RotterdamP5D0/2005/NL | 2005 | Netherlands | 3 | 22 |
| GU292851 | Hu/GII.P21-GII.3/RUS/Novosibirsk/Nsk-H677/2004 | 2004 | Russia | 3 | 28 |
| GU980585 | CBNU1 | 2006 | South Korea | 3 | 33 |
| JN899244 | Hu/GII.3/Glastonbury1164/2004/USA | 2004 | USA | 3 | Barclay, L., et al |
| KC464324 | Hu/GII.3/01-13/477/2001/AU | 2001 | Australia | 3 | 26 |
| KC464325 | Hu/GII.3/02-13/424cons/2002/AU | 2002 | Australia | 3 | 26 |
| KC464495 | Hu/GII.3/CGMH36/2010/TW | 2010 | Taiwan | 3 | 34 |
| KF006265 | TCH-104 | 2002 | USA | 3 | 35 |
| KF895848 | Hu/GII.P12-GII.3/RUS/Smolensk/S12-106/2012 | 2012 | Russia | 3 | 19 |
| KF895859 | Hu/GII.P12-GII.3/RUS/Krasnoyarsk/K12-32/2012 | 2012 | Russia | 3 | 19 |
| KF931180 | Hu/GII.P21-GII.3/RUS/Novosibirsk/Nsk-H382/2004 | 2004 | Russia | 3 | 28 |
| KF931234 | Hu/GII.P21-GII.3/RUS/Novosibirsk/Nsk-1763/2007 | 2007 | Russia | 3 | 28 |
| KF931243 | Hu/GII.P21-GII.3/RUS/Novosibirsk/Nsk-1818/2007 | 2007 | Russia | 3 | 28 |
| KF944203 | Hu/GII.P12-GII.3/RUS/Novosibirsk/Nsk-N3355/2011 | 2011 | Russia | 3 | 19, 28 |
| KF944227 | Hu/GII.P12-GII.3/RUS/Novosibirsk/Nsk-N4150/2012 | 2012 | Russia | 3 | 19, 28 |
| KF944232 | Hu/GII.P12-GII.3/RUS/Novosibirsk/Nsk-N4370/2012 | 2012 | Russia | 3 | 19, 28 |

**Supplementary Table S1** (continued). Strains used in this study (GII.3 genotype).

| GenBank accession No. | Strain | Collected year | Country | Cluster | References or authorship |
| --- | --- | --- | --- | --- | --- |
| KJ499441 | GII/Hu/HKG/2013/GII.3/CUHK-NS-193 | 2013 | China | 3 | Chan, M.C.W., et al |
| KJ499442 | GII/Hu/HKG/2013/GII.3/CUHK-NS-201 | 2013 | China | 3 | Chan, M.C.W., et al |
| KJ499444 | GII/Hu/HKG/2014/GII.3/CUHK-NS-227 | 2014 | China | 3 | Chan, M.C.W., et al |
| KJ499445 | GII/Hu/HKG/2014/GII.3/CUHK-NS-232 | 2014 | China | 3 | Chan, M.C.W., et al |
| KT732274 | 15-DD-3/2015/GII.3 | 2015 | Taiwan | 3 | Wu, F.-T., et al |
| KX989464 | Hu/016B01/ZS/GD/CHN/2016 | 2016 | China | 3 | Shi, W., et al |
| KX989465 | Hu/016B02/ZS/GD/CHN/2016 | 2016 | China | 3 | Shi, W., et al |
| KX989466 | Hu/016B03/ZS/GD/CHN/2016 | 2016 | China | 3 | Shi, W., et al |
| KX989467 | Hu/016B04/ZS/GD/CHN/2016 | 2016 | China | 3 | Shi, W., et al |
| KX989468 | Hu/016B05/ZS/GD/CHN/2016 | 2016 | China | 3 | Shi, W., et al |
| KY348697 | Hu/Guangzhou/GZ2013-L20/CHN/2013 | 2013 | China | 3 | 36 |
| KY348698 | Hu/Guangzhou/GZ2014-L304/CHN/2014 | 2014 | China | 3 | 36 |
| KY406922 | Hu/JC068/ZS/GD/CHN/2015 | 2015 | China | 3 | Shi, W., et al |
| KY406923 | Hu/JC050/ZS/GD/CHN/2015 | 2015 | China | 3 | Shi, W., et al |
| KY406924 | Hu/JC084/ZS/GD/CHN/2015 | 2015 | China | 3 | Shi, W., et al |
| KY406925 | Hu/JC020/ZS/GD/CHN/2015 | 2015 | China | 3 | Shi, W., et al |
| KY406926 | Hu/JC095/ZS/GD/CHN/2015 | 2015 | China | 3 | Shi, W., et al |
| KY406927 | Hu/JC104/ZS/GD/CHN/2015 | 2015 | China | 3 | Shi, W., et al |
| KY406928 | Hu/JC141/ZS/GD/CHN/2016 | 2016 | China | 3 | Shi, W., et al |
| KY406929 | Hu/JC041/ZS/GD/CHN/2015 | 2015 | China | 3 | Shi, W., et al |
| KY406930 | Hu/JC039/ZS/GD/CHN/2015 | 2015 | China | 3 | Shi, W., et al |
| KY406931 | Hu/JC038/ZS/GD/CHN/2015 | 2015 | China | 3 | Shi, W., et al |
| KY406932 | Hu/JC046/ZS/GD/CHN/2015 | 2015 | China | 3 | Shi, W., et al |
| KY406933 | Hu/JC031/ZS/GD/CHN/2015 | 2015 | China | 3 | Shi, W., et al |
| KY406934 | Hu/JC113/ZS/GD/CHN/2015 | 2015 | China | 3 | Shi, W., et al |
| KY406935 | Hu/JC115/ZS/GD/CHN/2016 | 2016 | China | 3 | Shi, W., et al |
| KY406936 | Hu/JC118/ZS/GD/CHN/2016 | 2016 | China | 3 | Shi, W., et al |
| KY406937 | Hu/JC111/ZS/GD/CHN/2015 | 2015 | China | 3 | Shi, W., et al |
| KY406938 | Hu/JC105/ZS/GD/CHN/2015 | 2015 | China | 3 | Shi, W., et al |
| KY406939 | Hu/JC103/ZS/GD/CHN/2015 | 2015 | China | 3 | Shi, W., et al |
| KY406940 | Hu/JC210/ZS/GD/CHN/2016 | 2016 | China | 3 | Shi, W., et al |
| KY406941 | Hu/JC155/ZS/GD/CHN/2016 | 2016 | China | 3 | Shi, W., et al |
| KY406942 | Hu/JC140/ZS/GD/CHN/2016 | 2016 | China | 3 | Shi, W., et al |
| KY406943 | Hu/JC135/ZS/GD/CHN/2016 | 2016 | China | 3 | Shi, W., et al |
| KY406944 | Hu/JC164/ZS/GD/CHN/2016 | 2016 | China | 3 | Shi, W., et al |
| KY406945 | Hu/JC259/ZS/GD/CHN/2016 | 2016 | China | 3 | Shi, W., et al |
| KY406946 | Hu/JC161/ZS/GD/CHN/2016 | 2016 | China | 3 | Shi, W., et al |
| KY406947 | Hu/JC159/ZS/GD/CHN/2016 | 2016 | China | 3 | Shi, W., et al |
| KY407172 | Hu/013F01/ZS/GD/CHN/2016 | 2013 | China | 3 | Shi, W., et al |
| KY407173 | Hu/013F02/ZS/GD/CHN/2016 | 2013 | China | 3 | Shi, W., et al |
| KY407174 | Hu/013F03/ZS/GD/CHN/2016 | 2013 | China | 3 | Shi, W., et al |
| KY407175 | Hu/013F04/ZS/GD/CHN/2016 | 2013 | China | 3 | Shi, W., et al |
| KY407176 | Hu/015G01/ZS/GD/CHN/2016 | 2015 | China | 3 | Shi, W., et al |
| KY407177 | Hu/015G02/ZS/GD/CHN/2016 | 2015 | China | 3 | Shi, W., et al |
| KY407178 | Hu/015G03/ZS/GD/CHN/2016 | 2015 | China | 3 | Shi, W., et al |
| KY407179 | Hu/015G04/ZS/GD/CHN/2016 | 2015 | China | 3 | Shi, W., et al |
| KY407180 | Hu/015G05/ZS/GD/CHN/2016 | 2015 | China | 3 | Shi, W., et al |
| KY407190 | Hu/015J01/ZS/GD/CHN/2016 | 2015 | China | 3 | Shi, W., et al |
| KY407191 | Hu/015J02/ZS/GD/CHN/2016 | 2015 | China | 3 | Shi, W., et al |
| KY407192 | Hu/015J03/ZS/GD/CHN/2016 | 2015 | China | 3 | Shi, W., et al |
| KY407193 | Hu/015J04/ZS/GD/CHN/2016 | 2015 | China | 3 | Shi, W., et al |
| KY407194 | Hu/015J05/ZS/GD/CHN/2016 | 2015 | China | 3 | Shi, W., et al |
| KY407195 | Hu/016A01/ZS/GD/CHN/2016 | 2016 | China | 3 | Shi, W., et al |
| KY407196 | Hu/016A02/ZS/GD/CHN/2016 | 2016 | China | 3 | Shi, W., et al |

**Supplementary Table S1** (continued). Strains used in this study (GII.3 genotype).

| GenBank accession No. | Strain | Collected year | Country | Cluster | References or authorship |
| --- | --- | --- | --- | --- | --- |
| KY407197 | Hu/016A03/ZS/GD/CHN/2016 | 2016 | China | 3 | Shi, W., et al |
| KY407198 | Hu/016A04/ZS/GD/CHN/2016 | 2016 | China | 3 | Shi, W., et al |
| KY767664 | Hu/GII.3/3-28/2015/HNZZ/CHN | 2015 | China | 3 | Zheng, L., et al |
| KY905334 | Hu/GII.P12_GII.3/QLDB207/2016/AU | 2016 | Australia | 3 | Lun, J.H. and White, P.A. |
| LC035072 | Hu/GII/14-2397/Tokyo/2014/JPN | 2014 | Japan | 3 | Mori, K. |
| LC035073 | Hu/GII/14-2523/Tokyo/2014/JPN | 2014 | Japan | 3 | Mori, K. |
| LC036561 | Hu/GII/14-3170/Tokyo/2014/JPN | 2014 | Japan | 3 | Mori, K. |
| LC101823 | GII/Hu/Jp/2007/GII.3/Kod1 | 2007 | Japan | 3 | Seto, Y., et al |
| LC133339 | Hu/GII.3/OsakaJA8/2014/JP | 2014 | Japan | 3 | Sakon, N., et al |
| LC133343 | Hu/GII.3/OsakaSB4/2014/JP | 2014 | Japan | 3 | Sakon, N., et al |
| MG892910 | Hu/GII.P12-GII.3/RUS/Novosibirsk/NS16-A651/2016 | 2016 | Russia | 3 | Zhirakovskaya, E. and Tikunova, N. |
| MG892911 | Hu/GII.P12-GII.3/RUS/Novosibirsk/NS16-A675/2016 | 2016 | Russia | 3 | Zhirakovskaya, E. and Tikunova, N. |
| MG892915 | Hu/GII.P12-GII.3/RUS/Novosibirsk/NS16-A768/2016 | 2016 | Russia | 3 | Zhirakovskaya, E. and Tikunova, N. |
| MG892920 | Hu/GII.P12-GII.3/RUS/Novosibirsk/NS16-A796/2016 | 2016 | Russia | 3 | Zhirakovskaya, E. and Tikunova, N. |
| MG892947 | Hu/GII.P16-GII.3/RUS/Novosibirsk/NS17-A863/2017 | 2017 | Russia | 3 | Zhirakovskaya, E. and Tikunova, N. |
| MG892949 | Hu/GII.P12-GII.3/RUS/Novosibirsk/NS17-A929/2017 | 2017 | Russia | 3 | Zhirakovskaya, E. and Tikunova, N. |
| MG892950 | Hu/GII.P12-GII.3/RUS/Novosibirsk/NS17-A928/2017 | 2017 | Russia | 3 | Zhirakovskaya, E. and Tikunova, N. |
| MG892951 | Hu/GII.P16-GII.3/RUS/Novosibirsk/NS17-A947/2017 | 2017 | Russia | 3 | Zhirakovskaya, E. and Tikunova, N. |
| MG892952 | Hu/GII.P16-GII.3/RUS/Novosibirsk/NS17-A996/2017 | 2017 | Russia | 3 | Zhirakovskaya, E. and Tikunova, N. |
| MG892953 | Hu/GII.P16-GII.3/RUS/Novosibirsk/NS17-A1029/2017 | 2017 | Russia | 3 | Zhirakovskaya, E. and Tikunova, N. |
| MG892956 | Hu/GII.P12-GII.3/RUS/Novosibirsk/NS17-A1335/2017 | 2017 | Russia | 3 | Zhirakovskaya, E. and Tikunova, N. |
| MK073886 | Hu/USA/2016/GII.P12-GII.3/AnnArbor-0027 | 2016 | USA | 3 | Soehnlen, M., et al |
| MN199033 | CAU140599 | 2014 | South Korea | 3 | 37 |

References

1. Jiang, X., Wang, M., Wang, K., Estes, M.K., 1993. Sequence and genomic organization of Norwalk virus. Virology 195 (1), 51-61.
2. Lew, J.F., Kapikian, A.Z., Valdesuso, J., Green, K.Y., 1994. Molecular characterization of Hawaii virus and other Norwalk-like viruses: evidence for genetic polymorphism among human caliciviruses. J. Infect. Dis. 170 (3), 535-542.
3. Green, S.M., Lambden, P.R., Caul, E.O., Ashley, C.R., Clarke, I.N., 1995. Capsid diversity in small round-structured viruses: molecular characterization of an antigenically distinct human enteric calicivirus. Virus Res. 37 (3), 271-283.
4. Green, S.M., Dingle, K.E., Lambden, P.R., Caul, E.O., Ashley, C.R., Clarke, I.N., 1994. Human enteric Caliciviridae: a new prevalent small round-structured virus group defined by RNA-dependent RNA polymerase and capsid diversity. J. Gen. Virol. 75 (Pt 8), 1883-1888.
5. Green, J., Vinje, J., Gallimore, C.I., Koopmans, M., Hale, A., Brown, D.W., Clegg, J.C., Chamberlain, J., 2000. Capsid protein diversity among Norwalk-like viruses. Virus Genes 20 (3), 227-236.
6. Katayama, K., Shirato-Horikoshi, H., Kojima, S., Kageyama, T., Oka, T., Hoshino, F., Fukushi, S., Shinohara, M., Uchida, K., Suzuki, Y., Gojobori, T., Takeda, N., 2002. Phylogenetic analysis of the complete genome of 18 Norwalk-like viruses. Virology 299 (2), 225-239.
7. Ando, T., Monroe, S.S., Noel, J.S., Glass, R.I., 1997. A one-tube method of reverse transcription-PCR to efficiently amplify a 3-kilobase region from the RNA polymerase gene to the poly(A) tail of small round-structured viruses (Norwalk-like viruses). J. Clin. Microbiol. 35 (3), 570-577.
8. Jiang, X., Zhong, W.M., Farkas, T., Huang, P.W., Wilton, N., Barrett, E., Fulton, D., Morrow, R., Matson, D.O., 2002. Baculovirus expression and antigenic characterization of the capsid proteins of three Norwalk-like viruses. Arch. Virol. 147 (1), 119-130.
9. Sugieda, M., Nagaoka, H., Kakishima, Y., Ohshita, T., Nakamura, S., Nakajima, S., 1998. Detection of Norwalk-like virus genes in the caecum contents of pigs. Arch. Virol. 143 (6), 1215-1221.
10. Kobayashi, S., Sakae, K., Suzuki, Y., Ishiko, H., Kamata, K., Suzuki, K., Natori, K., Miyamura, T., Takeda, N., 2000. Expression of recombinant capsid proteins of chitta virus, a genogroup II Norwalk virus, and development of an ELISA to detect the viral antigen. Microbiol. Immunol. 44 (8), 687-693.
11. Zheng, D.P., Ando, T., Fankhauser, R.L., Beard, R.S., Glass, R.I., Monroe, S.S., 2006. Norovirus classification and proposed strain nomenclature. Virology 346 (2), 312-323.
12. Wang, Q.H., Han, M.G., Cheetham, S., Souza, M., Funk, J.A., Saif, L.J., 2005. Porcine noroviruses related to human noroviruses. Emerging Infect. Dis. 11 (12), 1874-1881.
13. Iritani, N., Kaida, A., Kubo, H., Abe, N., Goto, K., Ogura, H., Seto, Y., 2010. Molecular epidemiology of noroviruses detected in seasonal outbreaks of acute nonbacterial gastroenteritis in Osaka City, Japan, from 1996-1997 to 2008-2009. J. Med. Virol. 82 (12), 2097-2105.
14. Green, K.Y., Belliot, G., Taylor, J.L., Valdesuso, J., Lew, J.F., Kapikian, A.Z., Lin, F.Y., 2002. A predominant role for Norwalk-like viruses as agents of epidemic gastroenteritis in Maryland nursing homes for the elderly. J. Infect. Dis. 185 (2), 133-146.
15. Symes, S.J., Gunesekere, I.C., Marshall, J.A., Wright, P.J., 2007. Norovirus mixed infection in an oyster-associated outbreak: an opportunity for recombination. Arch. Virol. 152 (6), 1075-1086.
16. Boon, D., Mahar, J.E., Abente, E.J., Kirkwood, C.D., Purcell, R.H., Kapikian, A.Z., Green, K.Y., Bok, K., 2011. Comparative evolution of GII.3 and GII.4 norovirus over a 31-year period. J. Virol. 85 (17), 8656-8666.
17. Rackoff, L.A., Bok, K., Green, K.Y. and Kapikian, A.Z., 2013. Epidemiology and evolution of rotaviruses and noroviruses from an archival WHO Global Study in Children (1976-79) with implications for vaccine design. PLoS One. 8 (3), e59394.
18. Lindesmith, L.C., Debbink, K., Swanstrom, J., Vinjé, J., Costantini, V., Baric, R.S., Donaldson, E.F., 2012. Monoclonal antibody-based antigenic mapping of norovirus GII.4-2002. J. Virol. 86 (2), 873-883.
19. Zhirakovskaia, E., Tikunov, A., Tymentsev, A., Sokolov, S., Sedelnikova, D., Tikunova, N., 2019. Changing pattern of prevalence and genetic diversity of rotavirus, norovirus, astrovirus, and bocavirus associated with childhood diarrhea in Asian Russia, 2009-2012. Infect. Genet. Evol. 67, 167-182.
20. Johnson, J.A., Parra, G.I., Levenson, E.A., Green, K.Y., 2017. A large outbreak of acute gastroenteritis in Shippensburg, Pennsylvania, 1972 revisited: evidence for common source exposure to a recombinant GII.Pg/GII.3 norovirus. Epidemiol. Infect. 145 (8), 1591-1596.
21. Lew, J.F., Petric, M., Kapikian, A.Z., Jiang, X., Estes, M.K., Green, K.Y., 1994. Identification of minireovirus as a Norwalk-like virus in pediatric patients with gastroenteritis. J. Virol. 68 (5), 3391-3396.
22. Siebenga, J.J., Beersma, M.F., Vennema, H., van Biezen, P., Hartwig, N.J., Koopmans, M., 2008. High prevalence of prolonged norovirus shedding and illness among hospitalized patients: a model for in vivo molecular evolution. J. Infect. Dis. 198 (7), 994-1001.
23. Eden, J.S., Bull, R.A., Tu, E., McIver, C.J., Lyon, M.J., Marshall, J.A., Smith, D.W., Musto, J., Rawlinson, W.D., White, P.A., 2010. Norovirus GII.4 variant 2006b caused epidemics of acute gastroenteritis in Australia during 2007 and 2008. J. Clin. Virol. 49 (4), 265-271.
24. Zhirakovskaya, E.V., Tikunov, A.Y., Kurilshchikov, A.M., Demina, A.V., Pokrovskaya, I.V., Sheronova, O.B., Pozdnyakova, L.L., Netesov, S.V., Tikunova, N.V., 2013. The etiological structure of acute enteric infections in adults in Novosibirsk. Infect. Dis. 11 (2), 31-37.
25. Xue, L., Wu, Q., Dong, R., Kou, X., Li, Y., Zhang, J., Guo, W., 2013. Genetic analysis of noroviruses associated with sporadic gastroenteritis during winter in Guangzhou, China. Foodborne Pathog. Dis. 10 (10), 888-895.
26. Mahar, J.E., Bok, K., Green, K.Y., Kirkwood, C.D., 2013. The importance of intergenic recombination in norovirus GII.3 evolution. J. Virol. 87 (8), 3687-3698.
27. Huo, Y., Cai, A., Yang, H., Zhou, M., Yan, J., Liu, D., Shen, S., 2014. Complete nucleotide sequence of a norovirus GII.4 genotype: evidence for the spread of the newly emerged pandemic Sydney 2012 strain to China. Virus Genes 48 (2), 356-360.
28. Zhirakovskaia, E.V., Tikunov, A.Y., Bodnev, S.A., Klemesheva, V.V., Netesov, S.V., Tikunova, N.V., 2015. Molecular epidemiology of noroviruses associated with sporadic gastroenteritis in children in Novosibirsk, Russia, 2003-2012. J. Med. Virol. 87 (5), 740-753.
29. Cotten, M., Petrova, V., Phan, M.V., Rabaa, M.A., Watson, S.J., Ong, S.H., Kellam, P., Baker, S., 2014. Deep sequencing of norovirus genomes defines evolutionary patterns in an urban tropical setting. J. Virol. 88 (19), 11056-11069.
30. Caddy, S.L., de Rougemont, A., Emmott, E., El-Attar, L., Mitchell, J.A., Hollinshead, M., Belliot, G., Brownlie, J., Le Pendu, J., Goodfellow, I., 2015. Evidence for human norovirus infection of dogs in the United Kingdom. J. Clin. Microbiol. 53 (6), 1873-1883.
31. Ruis, C., Roy, S., Brown, J.R., Allen, D.J., Goldstein, R.A., Breuer, J., 2017. The emerging GII.P16-GII.4 Sydney 2012 norovirus lineage is circulating worldwide, arose by late-2014 and contains polymerase changes that may increase virus transmission. PLoS ONE 12 (6), e0179572.
32. Brown, J.R., Roy, S., Shah, D., Williams, C.A., Williams, R., Dunn, H., Hartley, J., Harris, K., Breuer, J., 2018. Norovirus Transmission Dynamics in a Pediatric Hospital Using Full Genome Sequences. Clin. Infect. Dis. 68 (2), 222-228.
33. Yun, S.I., Kim, J.K., Song, B.H., Jeong, A.Y., Jee, Y.M., Lee, C.H., Paik, S.Y., Koo, Y., Jeon, I., Byun, S.J., Lee, Y.M., 2010. Complete genome sequence and phylogenetic analysis of a recombinant Korean norovirus, CBNU1, recovered from a 2006 outbreak. Virus Res. 152 (1-2), 137-152.
34. Tsai, C.N., Lin, C.Y., Lin, C.W., Shih, K.C., Chiu, C.H., Chen, S.Y., 2014. Clinical relevance and genotypes of circulating noroviruses in northern Taiwan, 2006-2011. J. Med. Virol. 86 (2), 335-346.
35. Kou, B., Crawford, S.E., Ajami, N.J., Czako, R., Neill, F.H., Tanaka, T.N., Kitamoto, N., Palzkill, T.G., Estes, M.K., Atmar, R.L., 2015. Characterization of cross-reactive norovirus-specific monoclonal antibodies. Clin. Vaccine Immunol. 22 (2), 160-167.
36. Xue, L., Wu, Q., Dong, R., Cai, W., Wu, H., Chen, M., Chen, G., Wang, J., Zhang, J., 2017. Comparative phylogenetic analyses of recombinant noroviruses based on different protein-encoding regions show the recombination-associated evolution pattern. Sci. Rep. 7 (1), 4976.
37. Seo, D.J., Jung, D., Jung, S., Ha, S.K., Ha, S.D., Choi, I.S., Myoung, J., Choi, C., 2018. Experimental miniature piglet model for the infection of human norovirus GII. J. Med. Virol. 90 (4), 655-662.

**Supplementary Table S2.** Evolutionary rates for each cluster.

|  | Evolutionary rates (95% HPD)  (substitutions/site/year) |
| --- | --- |
| All GII.3  (239 strains) | 4.82 × 10^−3^ (4.14 × 10^−3^ – 5.48 × 10^−3^) |
| Cluster 1  (25 strains) | 3.77 × 10^−3^ (2.61 × 10^−3^ – 4.94 × 10^−3^) |
| Cluster 2  (121 strains) | 5.29 × 10^−3^ (4.29 × 10^−3^ – 6.34 × 10^−3^) |
| Cluster 3  (93 strains) | 4.40 × 10^−3^ (3.24 × 10^−3^ – 5.56 × 10^−3^) |
